# Diagnosing Mechanisms of Decline and Planning for Recovery of an Endangered Brown Bear (*Ursus arctos*) Population

**DOI:** 10.1371/journal.pone.0007568

**Published:** 2009-10-28

**Authors:** Guillaume Chapron, Robert Wielgus, Pierre-Yves Quenette, Jean-Jacques Camarra

**Affiliations:** 1 Grimsö Wildlife Research Station, Department of Ecology, Swedish University of Agricultural Sciences, Riddarhyttan, Sweden; 2 Conservation Biology Division, Institute of Zoology, University of Bern, Bern, Switzerland; 3 Laboratoire d'Ecologie Animale, Faculté des Sciences, Université d'Angers, Campus de Belle-Beille, Angers, France; 4 Museum National d'Histoire Naturelle, Département Ecologie et Gestion de la Biodiversité, Paris, France; 5 Large Carnivore Conservation Laboratory, Department of Natural Resource Sciences, Washington State University, Pullman, Washington, United States of America; 6 Office National de la Chasse et de la Faune Sauvage, CNERA-PAD, Equipe Technique Ours - Réseau Ours Brun, Impasse de la Chapelle, Villeneuve de Rivière, France; 7 Office National de la Chasse et de la Faune Sauvage, CNERA-PAD, Equipe Technique Ours - Réseau Ours Brun, Pau, France; University of Pretoria, South Africa

## Abstract

**Background:**

The usual paradigm for translocations is that they should not take place in declining populations until the causes(s) of the decline has been reversed. This approach sounds intuitive, but may not apply in cases where population decline is caused by behavioral or demographic mechanisms that could only be reversed by translocation itself.

**Methodology/Principal Findings:**

We analyzed a decade of field data for Pyrenean brown bears (*Ursus arctos*) from two small populations: the growing Central population - created from a previous translocation and the endemic Western population - believed to be declining because of excessive human-caused mortality. We found that adult survival rates for both populations were as high as those observed for most other protected brown bear populations. However, the Western population had much lower reproductive success than the Central population. Adult breeding sex ratio was male-biased in the Western population and female-biased in the Central population. Our results exclude high anthropogenic mortality as a cause for population decline in the West but support low reproductive success, which could result from sexually selected infanticide induced by a male-biased adult sex ratio or inbreeding depression. Using a stochastic demographic model to compute how many bears should be released to ensure viability, we show that the Western population could recover provided adequate numbers of new females are translocated.

**Conclusions/Significance:**

We suggest that a translocation could take place, even if the decline has not yet been reversed, if the translocation itself removes the biological mechanisms behind the decline. In our case, the ultimate cause of low reproductive success remained unknown (infanticide or inbreeding), but our proposed translocation strategies should eliminate the proximate cause (low reproductive success) of the decline and ensure population recovery and viability.

## Introduction

The increased awareness of the public to conserve biodiversity has made possible a number of translocation programs to restore previously extirpated predator populations [1]. With translocation projects becoming increasingly well documented [2], it is now possible to draw general rules and identify key factors for project success. Miller et al. [3] reviewed biological and technical considerations for carnivore translocations and identified a set of critical biological factors, including animal selection, genetics, demography, behavior, health, and habitat. They also stressed that translocation should include a feasibility study to address important questions such as whether or not the causes of population decline or extirpation have been eliminated. This general rule to release animals only if demographic parameters are favorable is widely accepted and explicitly stipulated in the IUCN guidelines for reintroductions [4]. Yet, little research has focused on improving translocation success by manipulating behavioral and demographic mechanisms (see however [5]). In this paper, we focus on the Pyrenean brown bear (*Ursus arctos*) population to illustrate how translocations can be designed to eliminate the biological mechanisms of decline and attain population recovery in a situation that seems desperate.

The brown bear population in France disappeared from most of the country during the 20^th^ century [6], [7] and now only survives in the Pyrénées mountain range. In 1995, only 5 bears remained in the Western part of the Pyrénées range. An experimental translocation of two females and one male originating from Slovenia was carried out in 1996–97 in the Central part of the range [8]. Both populations are separated by a few hundred kilometers and share the same favorable ecological habitat. Since then, despite one released female being killed, the newly created Central population has grown [9]. On the contrary, the endemic Western population has continued to decline and lost its last female in November 2004 during a hunting accident. The main impediment to population recovery was believed to be high anthropogenic mortality [10], [11], [12] and there is a continuous debate as to whether additional transplant augmentations should even proceed. With the apparently high human-caused mortality, translocating new bears seemed hopeless and very difficult to defend politically. An alternative explanation for the recent population decline in the Pyrénées has been suggested by Wielgus et al. [13]. They proposed that a male-biased sex ratio in such a small population could result in increased sexually selected infanticide (low cub survival [14], [15]) and sexual segregation (low cub production [16], [17], [18], [19]). McLellan [20] similarly suggested that male-biased sex ratios could result in sexually selected infanticide in North American grizzly bears. The Western population could also suffer from isolation and inbreeding, resulting in low reproduction [21]. If population growth in the Pyrénées was limited by low reproduction, not high anthropogenic mortality, removing the cause of population decline recommended *before* any translocation could in fact only be achieved by a translocation itself to increase reproductive success by re-equilibrating the sex ratio and/or reducing inbreeding depression.

In this paper, we analyze field data and estimate demographic parameters for the Western and Central Pyrénées brown bear populations for the period 1993–2005. As our analysis is based on a comparison of Western and Central populations, we do not consider data after 2005, when the Western population went functionally extinct (no females remained). We try to identify the ultimate and proximate cause(s) of decline in the Western population and compute the numbers of transplant bears required - if possible - to achieve population viability.

## Methods

### Monitoring of the bear population

Brown bears were monitored for births and deaths from 1993 to 2005 by a combination of visual sightings, track identifications, genotyping, camera-trapping, and radio-telemetry by resident project biologists over 5000 km^2^ in the Pyrénées mountains of France [9], [22], [23], [24], [25], [26], [27], [28]. Ten bears were monitored in the Western Pyrénées from 1993 to 2005 (13 years) for a total of 53 bear-years. Sixteen bears were monitored in the Central Pyrénées from 1995 to 2005 (11 years) for a total of 67 bear-years. Easy access and intensive monitoring allowed identification of all bears in the populations, which were individually known and whose lives were widely covered by the local media. In addition, biologists determined in spring how many cubs were born (or left the den) by intensively camera-trapping the breeding female home ranges, systematically prospecting for tracks and analyzing their sizes, and genotyping hairs trapped on rubber pads sprayed with turpentine oil.

### Demographic parameter estimates

We defined age classes as cubs (0–1 year of age), yearlings (1–2 years of age), sub-adults (2–4 years of age), and reproductively successful adults (4+ years of age). Observed age at first successful birth by females defined the adult class of 4+ years. Mean litter size was estimated as the average number of newborns observed during early summer (April–July) in each successful litter. Mean birth interval was estimated as the average number of years between successful births for each female. Mean annual maternity rate *Mx* was estimated as the average number of cubs/adult female/year [29]. Mean annual recruitment rate (*Rec*) to 1 year of age was estimated as the arithmetic average of the product of annual *Mx* and mean cub survival (Rec  =  Mx*Sc). Standard deviations (SD) of annual *Mx* and *Rec* were calculated for a measure of annual environmental stochasticity [30], [31]. We estimated the annual mortality rate of cubs using the Mayfield staggered entry method [32] whereby we divided the number of cubs that died in their first year of life by the total number of observed cub-years for each area. Survival rate of cubs (*Sc*) was the reciprocal of cub mortality rate. We estimated the Mayfield mortality and survival rates of yearlings (*Sy*), sub-adults (*Ss*), and adults (*Sa*) in the same manner. We used the Heisey & Fuller [32] method and not the Kaplan-Meirer [33] or Pollock et al. [34] method for this first phase of our analyses because the Kaplan-Meier method can yield highly biased results when observed numbers of animals are very small (e.g., for each sex/age class) during any portion of a time series [35]. Because survival of very small numbers of yearlings and sub-adults were 1.00 (see Results), those age classes were pooled in order to increase the numbers of animals for subsequent analyses. Survival rate of the larger pooled age class was then estimated using the staggered entry, Kaplan-Meier product estimator [36]. Right-censored animals that disappeared entirely from the study region (never to be seen again) were considered effectively dead, because of intensive monitoring of all bears in the study region and because animals that left the study area could not contribute to population growth. Standard deviations (SD) of annual survival were calculated to estimate annual environmental stochasticity in survival. Annual adult sex ratios (*SR*) were estimated as the number of reproductive (4+ years old) males/reproductive (4+ years old) females. Mean sex ratios were the arithmetic averages summed over years. SD of *SR* was used as a measure of environmental stochasticity in sex ratio. We did not construct 95% confidence intervals of measurement error around any of the demographic parameter estimates or conduct statistical tests for differences among mean rates between areas - because there was no sampling error associated with any of the parameter estimates. As the bears present within the populations were monitored in this way there could be no sampling variability or error in these known, closed populations [31]. Process error [36] was very small, i.e. differences between modeled and observed growth rates were 0.00 to 0.01 (see Results).

### Sex ratio and reproductive success

We conducted a linear regression to estimate the relationships between annual *SR* and annual *Mx* and *Rec*. The time series contained a majority of zeros for *Mx* and *Rec*, because of the extended birth intervals in brown bears. Because these zeros would render regression analyses ineffective, we transformed the annual estimates of *Mx, Rec*, and *SR* into 3-year running averages (the normal birth interval) to eliminate most of the zeros. Because sex ratio was very strongly confounded with area (see Results), we followed the regression analyses with an ANCOVA to try to account for the combined effects of area and sex ratio.

### Population modeling

We constructed an age-structured female matrix model using ULM software [37]. In the model, bears could be cubs (0–1 year of age), pooled sub-adults (1–4 years of age) or breeders (4+ year old) and demographic parameters in the models were survival and fecundity rates previously computed. Age at reproductive senescence was set at 25 years – the norm for brown bear populations [38]. We used this model to compute the asymptotic growth rate λ of each population [39]. We compared the asymptotic growth rate λ with the observed annual growth rate *Ro* as (*Nt*+*x*/*Nt*)^1/*x*^ where *N* is the number of animals and *x* is the number of years in the time series [40].

Keeping the same population structure, we developed a 2-sex stochastic model and the population was divided into the same classes that could either be males or females. Females in the model could reproduce as soon as at least one adult male was present in one of the population. We assumed that the two populations were demographically independent, because there was a complete lack of female movement between populations and since brown bear females are usually philopatric [41], [42]. Our stochastic model included both demographic and environmental stochasticity. Demographic stochasticity was applied to both survival and fecundity. Class survival followed a binomial distribution (i.e. a random number was drawn from a Bernoulli trial with class survival as a parameter and a bear survived if the result was 1 and died if it was 0). Fecundity followed a Poisson distribution. Environmental stochasticity was estimated as annual variability or standard deviations in the survival rates and recruitment/fecundity rates observed in the time-series. A population was classified as extinct once all sex and age classes were empty. We used this definition for extinction, not the usual definition of 0 females, to allow cases of augmentation where only individuals of the same sex remained [11]. We used the stochastic model to compute the extinction probabilities for the two populations associated with several translocation strategies under different population scenarios.

### Simulations of translocation strategies

Probabilities of extinction (*Pext*) of both Western and Central populations were computed for 30 years (the maximum lifespan of bears). We did not run longer simulations because social, economic and political situations, which affect population parameters, will likely have changed in a few decades. Bear translocations of *n_f_* females and *n_m_* males were modeled as single events, i.e. *n_f_* + *n_m_* individuals simultaneously joined the population. We assumed all transplanted bears would be sexually mature (e.g. 4 years of age). All computations were Monte Carlo simulations of 1000 runs. We ran simulations for the Western population under two separate scenarios. The first scenario assumed that recruitment or fecundity rate was influenced by sex ratio (*SR*) of breeders. In this case, we defined recruitment rate *Fx* as a negatively correlated variable with breeder sex ratio: *Fx* = −0.23*SR+0.738 (see Results). Hence, fecundity was a dynamically changing parameter during the simulation. The second scenario assumed that the previously observed low recruitment/fecundity rate observed in the West (*Fx* = 0.17±0.25) was due to inbreeding. For this inbreeding scenario, new in-coming females would have the higher rates that we found in the Central population, originated from reintroduced Slovenian females: (*Fx* = 0.55±0.60). This assumption is possible because no potentially inbred females remained in the West (the last one was killed in 2004).

We ran simulations for the Central population under two separate scenarios. The first is the sex ratio scenario, as was done for the Western population. For the second scenario, because potential inbreeding was low in this population, we simply kept the high fecundity rate of this population. For each of our scenarios, we evaluated the influence of *n_f_* and *n_m_* (varying from 0 to 10) on the probability of extinction for both populations and selected the smallest combination of *n_f_* and *n_m_* under each of the scenarios that led to an extinction probability smaller than 3%. We chose 3% on 30-year simulations to match the IUCN definition of population viability as having an extinction probability less than 10% during 100 years [43]. Those numbers of transplants are to be considered as strict minima under which population viability is likely to fail - and should not be considered as absolute targets.

## Results

### Demographic parameters for 1993–2005

Reproductive success was much higher in the Central population compared to the Western population. Mean litter size was 1.86±0.69 (*N* = 7) in the Central vs. 1.0±0.0 (*N* = 4) in the West. Mean birth interval was 2.0±0.82 years (*N* = 4) in the Central vs. 3.0±1.0 years (*N* = 3) in the West. Mean maternity rate *Mx* was more than twice as high in the Central population: 0.72/year±0.79 vs. 0.33/year±0.49. Mean recruitment rate was three times as high in the Central population: 0.55±0.60 vs. 0.17±0.25. Mayfield survival rates were *Sc* = 0.77±0.11 for cubs, *Sy* = 0.90±0.09 for yearlings, *Ss* = 1.00 for sub-adults, and *Sa* = 0.97±0.03 for adults in the Central population. Corresponding rates for the Western population were *Sc* = 0.50±0.25, *Sy* = 1.0±0.0, *Ss* = 1.0±0.0, and *Sa* = 0.91±0.04. Kaplan Meier survival rates for pooled *Sy, Ss*, and *Sa* were similar between populations: 0.94±0.11 in the Central vs. 0.91±0.12 in the West. The only substantive difference in survival between areas was for cubs (0.77 in the Central vs. 0.50 in the West). Average annual sex ratio was heavily skewed towards males in the West (2.22±0.65) and females in the Central population (0.69±0.20).

### Sex ratio and reproductive performance

**Figure 1:**
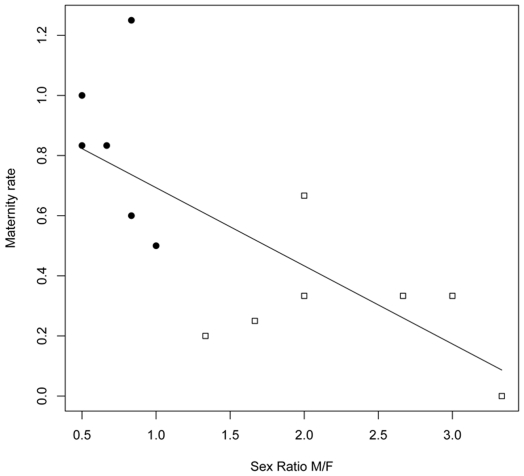
Relation between maternity rate and adult sex ratio. There is a statistically significant negative relationship between 3-year average maternity rate *Mx* and 3-year average sex ratio *SR* (*Mx* = −0.26*SR+0.95) for brown bears in the Central (black circles) and Western populations (empty squares).

**Figure 2:**
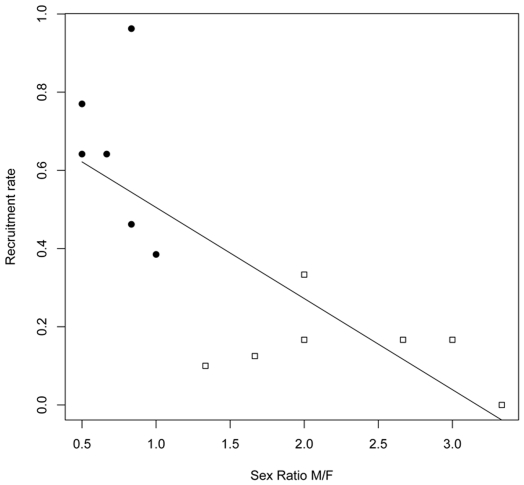
Relation between recruitment rate and adult sex ratio. There is a statistically significant negative relationship between 3-year average recruitment rate *Rec* and 3-year average sex ratio *SR* (*Rec* = −0.23*SR+0.74) for brown bears in the Central (black circles) and Western populations (empty squares).

There were statistically significant negative relationships between 3-year average maternity rates and 3-year average sex ratios (R^2^ = 0.57, *p*<0.001, Figure 1), and 3-year average recruitment rates and 3-year average sex ratios (R^2^ = 0.64, *p*<0.001, Figure 2). As sex ratios increased towards males, these two measures of female reproductive performance declined. However, these statistically significant negative relationships disappeared (*p*>0.10) when the area effect was incorporated into an ANCOVA.

### Population growth

The Central population was increasing rapidly while the Western population was declining. The asymptotic growth rates λ were 1.11 for the Central population and 0.95 for the Western population. The observed growth rates (*Ro*) were very close to λ at 1.11 for the Central population and 0.96 for the Western population.

### Simulations of translocations

**Figure 3:**
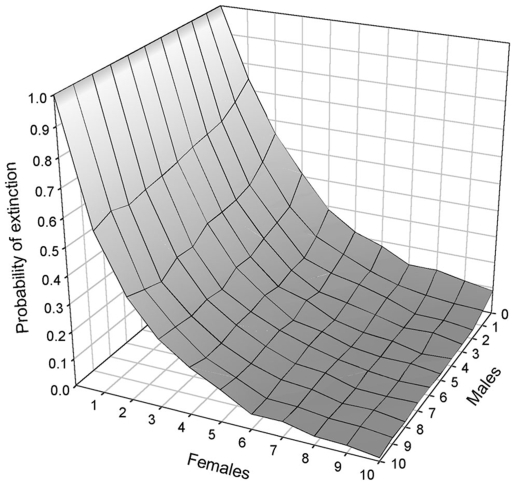
Probability of extinction for the Western population under the inbreeding scenario. Releasing several females drastically reduces the probability of extinction.

**Figure 4:**
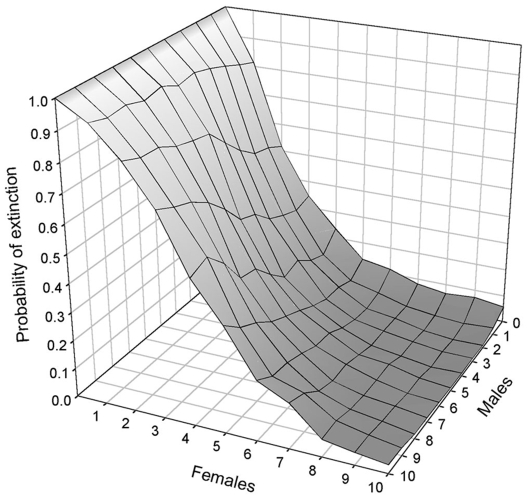
Probability of extinction for the Western population under the sex ratio scenario. For a given number of released females, releasing more than 2 males increases the probability of extinction by biasing the population adult sex ratio toward males.

The Western population had no remaining females (*Pext*  = 1) and obviously needed females to persist. However, the nature of the mechanism underlying the population decline had an effect on the numbers of bears required for transplant. Under the inbreeding scenario, 10 females and 3 males were required for a *Pext* <3% in 30 years (Figure 3). In this case, releasing females had a very strong effect on *Pext*. For example, with the release of a single female, we had *Pext*  = 0.74, but with 2 more transplants *Pext* dropped to 0.41. On the contrary, releasing males has a limited effect on *Pext*, even if several females were released. For example, with 3 transplant females (*Pext*  = 0.41), releasing 1 or 2 additional males decreased extinction probability only slightly (respectively *Pext*  = 0.32 and *Pext*  = 0.30). Under the sex ratio scenario, population recovery required at least 8 females and 1 male (Figure 4). Similar to the inbreeding scenario, female transplants had a strong effect on extinction probability, but on the contrary, releasing too many males actually increased *Pext*. The only exception is when a large number (>7) of females were released, in that case releasing a limited number of males (<3) decreased *Pext*. Numbers of transplants required for viability under both scenarios were much smaller in the Central population (3 females and 1 male). To summarize, we found that the minimum numbers of transplant bears required for recovery as of 2005, accounting for both inbreeding or sex ratio scenarios, were 10 females and 3 males for the Western population and 3 females and 1 male for the Central population.

## Discussion

### Demographic parameters for 1993–2005

Our data rejected the previously accepted “high mortality” hypothesis and supported the “low reproduction” hypothesis of recent population decline in the Pyrénées. Annual survival rates *Sp* of pooled yearlings, sub-adults, and adults in both populations (*Sp* = 0.91 and 0.94) were very similar to those calculated for other brown bear populations worldwide (mean *Sp* = 0.92, *N* = 7 [44]; mean *Sp* = 0.92, *N* = 11, [29]). It appears that the steps taken to reduce anthropogenic mortality by the French Government and local stakeholders from 1993 to 2005 have shown positive results compared to historical trends [45]. By contrast, there were large differences in reproductive success between the two populations, and the Western population was declining as a result (*λ* = 0.95 vs. 1.11). The measures of mean litter size and mean birth interval indicated relatively low reproductive performance in the West compared to most other populations [29], [38], [44]. Furthermore, the measures of maternity rate, cub survival, and recruitment rate in the West (*Mx* = 0.33, *Sc* = 0.50, *Rec* = 0.17) were much lower than in the Central population (*Mx* = 0.79, *Sc* = 0.77, *Rec* = 0.55), and lower than the ones reported in most other populations (mean *Rec* = 0.30, *N* = 7, [44]; mean *Rec* = 0.23, *N* = 11, [29]).

### Population growth

The Central population was growing from 1993 to 2005 at a rate (*λ* = 1.11, *Ro* = 1.11) very similar to that observed by Sæther et al. [46] and Swenson et al. [15] and is probably very close to the maximum growth rates for brown bears. The Western population was declining and its finite growth rate (*λ* = 0.95) was very close to the observed growth rate (*Ro* = 0.96). The rates were identical (*λ* = 1.11, *Ro* = 1.11) in the Central population. Such close correspondence between *λ* and *Ro* gives us confidence in the validity of the demographic parameters (*Mx*, *Sx*, *Sp*), used to estimate *λ* [36], and the extremely large differences in *Mx* and *Sc* were the only possible explanations for the large differences in *λ* and *Ro* between populations.

### Mechanism of decline

The very low levels of cub production, cub survival, and recruitment in the Western population are of critical importance for the conservation of this population. The first explanatory hypothesis we can retain is sexually selected infanticide (SSI, see [47] for a review) which long-term data reveals does occur in brown bears [14], [15], [47], [48], [49], [50]. SSI could affect population viability via reduced fecundity [13]. Previous research on other large carnivores also revealed SSI can have severe demographic consequences. In Israel, a highly male biased (4M/1F) population of leopards (*Panthera pardus*) around the Dead Sea is believed to have collapsed due to repeated cub death related to SSI [51], [52], [53]. Wielgus and Bunnell [16], [19] demonstrated a link between sex ratio, SSI, and litter size in brown bears. Swenson et al. [15], [50] documented the negative effects of SSI on brown bear cub survival. In both studies, as putative (resident) fathers of cubs died, immigrant non-fathers moved in and either attempted to kill or killed unrelated cubs to induce estrous in the resident females – to maximize their own genetic fitness. SSI did not affect animals older than cubs because killing of such animals failed to hasten estrous [50]. The phenomenon of SSI does not always require the death of fathers, but is simply an exacerbated manifestation of intra-male sexual competition [13] and the small litters in the West could also be explained by the same mechanism. Wielgus and Bunnell [17], [18] showed that male-biased sex ratios were associated with female avoidance of potentially infanticidal males and sexual segregation of females into food-poor environments; the result was smaller litter sizes than would otherwise be the case [19].

We must, however, stress that the proposed negative correlations between sex ratio *SR* and *Mx, Sc*, and *Rec* are not explanatory or unequivocal. Although we suspect that the unbalanced sex ratio in the West may have been a contributing factor to the low reproductive performance – that assertion cannot be proved here. The observed negative relationship between sex ratio and reproductive performance in the linear regression disappeared, after first inserting an area effect into the ANCOVA, because variability in sex ratio was almost entirely confounded with area. The male-biased sex ratios were overwhelmingly observed in the West and the female biased sex ratios in the Central populations (Figures 1 and 2). As such, there was very little or no interspersion for sex ratio between areas and the area effect completely subsumed variation in sex ratio in the ANCOVA. Such statistical problems are typical of research on rare endangered populations, but the only solution – trying to replicate the study elsewhere – is unfeasible.

There are other equally plausible explanations for the low reproductive success in the West that also correspond to the statistically significant area effect in the ANOVA. For example, the small resident population in the West may have gone through a genetic bottleneck in the past, resulting in a high degree of inbreeding and associated low reproductive performance. Taberlet *et al.* [28] found that the level of genetic polymorphisms in the Western Pyrénées brown bears was very low and indicative of inbreeding depression. However, the levels of polymorphisms were no lower than the ones found in the Kodiak Island brown bear population in Alaska [54], which shows high reproductive performance [55]. Yet another possibility is that the Western population could suffer from poorer environmental conditions than the Central population, but there is absolutely no data to validate this assumption. On the contrary, the fact that before the 1995–1996 release of Slovenian bears, the only remaining individuals were located in the West is likely an indication of good habitat conditions. The Western and Central populations can be seen as two replicates of real world population experiment on a large carnivore species. Finally, a possible alternative is that the very few animals living in the West (but not in the Centre) just happened to go through a rare string of demographic stochastic bad luck for both *Mx* and *Sc* [30]. To summarize, the ultimate cause of the low reproductive performance cannot be assigned to a male-biased sex ratio, inbreeding depression, or demographic stochasticity. However, what is now known is that the two populations were not severely limited by unusually high anthropogenic mortality as previously believed. The proximate cause of the decline was very low reproductive success in the West.

### Simulations of translocation strategies

We computed minimal number of bears required for release, as of 2005, considering the two most plausible mechanisms of recent population decline (sex ratio or inbreeding effects). Our study revealed that the virtually extinct Western population would have required in 2005 a transplant of at least 13 bears to achieve viability. We also found that this recovery strategy depended on the mechanism responsible for the low reproductive success observed in the Western population. This population required 3M/10F under the inbreeding scenario but only 1M/8F under the sex ratio scenario - because skewing the sex ratio toward females via transplant may lead to higher fecundity rates. On the contrary, the Central population was not yet viable but just 4 bears (1M, 3F) were required, assuming demographic parameters remained the same after the releases. Since we cannot discriminate between the two scenarios (sex ratio or inbreeding), we provided a conservative minimum numbers of bears to release, which should ensure viability under either scenario. This explained the relatively large number of bears to be released in the Western population. IUCN guidelines for reintroduction [4] and others [3] stress that cause(s) of decline must be suppressed before releasing animals. Here, the exact cause(s) of decline remained unknown, but our proposed translocation strategies are intended to suppress the mechanism behind decline, whether caused by inbreeding or male biased sex ratio.

In 2006, the French Government released 5 Slovenian bears (1M and 4F) in the Central Pyrénées. While a minimum 3 female transplants were recommended for the Central population, releasing the remaining captured female in the West would have been risky because of the possibility of infanticide due to sex ratio effects, so she was also released in the Central area. Two of these released females in the Central population later died (one by falling from a cliff, and one from a car accident). The bear population is still being monitored and more recent data will be used to update our models for future conservation. As the Western population has no females, ensuring population recovery for this area requires that the French Government releases 13 bears. The fact that non-cub survival rates in both populations were relatively high from 1993 to 2005 reveals that bear/human cohabitation in France can be possible, and success of further translocations is likely – even in the Western population if efforts to further reduce anthropogenic mortality are actively pursued. Previous brown bear translocation programs in Europe have also been successful: in the Italian Alps for example, 10 bears were released between 1999 and 2002 during an augmentation program for a relict population, and cub production has since been high [56]. This suggests that further translocations in the Pyrénées should have a high probability of success.

Our study illustrates that quantitative demography and population modeling can be critical for program design and success [2]: while the Western population has a λ <1, we show it still could persist and recover provided adequate translocation strategies are implemented. A translocation could still take place because the translocation itself removes the biological mechanism (low reproductive success) behind the decline.
